# Exploring unconventional attributes of red blood cells and their potential applications in biomedicine

**DOI:** 10.1093/procel/pwae001

**Published:** 2024-01-25

**Authors:** Alkmini T Anastasiadi, Vasiliki-Zoi Arvaniti, Krystalyn E Hudson, Anastasios G Kriebardis, Constantinos Stathopoulos, Angelo D’Alessandro, Steven L Spitalnik, Vassilis L Tzounakas

**Affiliations:** Department of Biochemistry, School of Medicine, University of Patras, 26504 Patras, Greece; Department of Biochemistry, School of Medicine, University of Patras, 26504 Patras, Greece; Department of Pathology and Cell Biology, Columbia University Irving Medical Center, New York City, NY 10032, USA; Laboratory of Reliability and Quality Control in Laboratory Hematology (HemQcR), Department of Biomedical Sciences, School of Health & Caring Sciences, University of West Attica (UniWA), 12243 Egaleo, Greece; Department of Biochemistry, School of Medicine, University of Patras, 26504 Patras, Greece; Department of Biochemistry and Molecular Genetics, University of Colorado Anschutz Medical Campus, 13001 Aurora, CO, USA; Department of Pathology and Cell Biology, Columbia University Irving Medical Center, New York City, NY 10032, USA; Department of Biochemistry, School of Medicine, University of Patras, 26504 Patras, Greece

## We need to talk about red blood cells

Eukaryotic cells are defined as cells containing distinct nuclei and other membranous organelles. However, red blood cells (RBCs) lack a nucleus and organelles—perhaps to limit the generation of reactive oxygen species (Zhang et al., 2011)—and are filled with hemoglobin (Hb). This oxygen carrier is limited to the cytosol by the cell membrane and cytoskeleton, which maintain cellular integrity and deformability as RBCs pass through narrow capillaries (Gratzer, 1994) to perform their primary function of gas exchange. Moreover, in the absence of mitochondria, RBCs are believed to have a quite simple energy metabolism based on glucose catabolism (van Wijk and van Solinge, 2005). For years, RBCs were viewed as mere hemoglobin carriers and there were—and still are—discussions on whether they should really be considered as classic living cells. RBCs are considered as terminally differentiated cells based on the value of the half-cell reduction potential (E_hc_) of the glutathione disulfide (GSSG)/glutathione (GSH) couple, which is linked to the biological status of the cell (proliferation–differentiation–apoptosis), and has been proposed as a key parameter associated with fundamental cellular biology (Schafer and Buettner, 2001). Yet, due to significant variations in human populations, the reported RBC E_hc_ levels in several cases resemble those of apoptotic cells (van ‘t Erve et al., 2013). Even so, as Walter Gratzer stated, RBCs are “more red than dead” (Gratzer, 1984) and, as complemented by Tibor Greenwalt, “a cell doesn’t have to have a nucleus to be respected” (Greenwalt, 1995). In this context, we will deconstruct the RBC status quo by examining a series of “what ifs”.

## Unconventional RBC features

### What if RBCs are not so empty after all?

During terminal erythropoiesis, human RBC progenitors extrude their highly condensed nucleus to yield reticulocytes, which then lose their remaining organelles during maturation into RBCs (Mei et al., 2021). While retained in most adult vertebrates except for mammals (Claver and Quaglia, 2009), nucleated RBCs can be found in the circulation of human fetuses but disappear in neonates. Their existence in the peripheral blood of neonates or adults is indicative of pathophysiological conditions (Haase, 2013; Stachon et al., 2007). Additionally, some subpopulations of mature RBCs retain their mitochondria; this phenomenon was reported in several disorders, including sickle cell disease (SCD) (Jagadeeswaran et al., 2017; Moriconi et al., 2022), Rhett syndrome (Sbardella et al., 2021), and systemic lupus erythematosus (SLE) (Caielli et al., 2021). Despite being the minority of circulating RBCs, mitochondria-positive RBCs may have significant effects, having been linked to oxidative stress, lysis phenomena, and immune responses (Caielli et al., 2021; Esperti et al., 2023; Moriconi et al., 2022; Tumburu et al., 2021). The release into the circulation of immature RBC subpopulations, such as nucleated and mitochondria-retaining, is often observed when there is a high demand for RBC production in the bone marrow or when ineffective erythropoiesis occurs (Pikora et al., 2023). Moreover, mitochondrial DNA mutagenesis (Ahlqvist et al., 2015), as well as defects in the ubiquitin (Ub) proteasome system [e.g., in SLE (Caielli et al., 2021)], impair mitochondrial elimination in the erythroid lineage.

Mature RBCs, lacking a nucleus and organelles, were thought to rely on their finite proteome throughout their ~120-day circulatory lifespan. Unexpectedly, a recent study confirmed that mature RBCs retain an, albeit limited, ability to translate not only primarily globin transcripts but also other gene transcripts (e.g., Fos proto-oncogene, *FOS*; JunB proto-oncogene, *JUNB*; ZFP36 ring finger protein*, ZFP36*; ubiquitin B*, UBB*) (Kumar et al., 2022). Interestingly, the use of translation inhibitors highlighted the importance of this low-level translation in maintaining normal amounts of globin chains (Kumar et al., 2022). It should be noted that this work contained several validation steps to ensure that the observed translation took place in mature RBCs and not reticulocytes, but with regard to non-RBC-specific genes, the results are discussed with caution and should be further validated using extensively leukoreduced samples. RBCs are also equipped with post-translational machinery to respond actively to changes in their environment. Being one of the cell types with the most kinases and phosphatases, it is not surprising that the regulation of RBC cytoskeletal elements and, consequently, membrane flexibility depend upon phosphorylation events (Cilek et al., 2023). These modifications are also implicated in pathological states, as in SCD (Dzandu and Johnson, 1980). Finally, the presence of balanced protein turnover machinery renders the RBC protein life cycle complete. For example, mature RBCs have functional proteasomes, which are hypothesized to be mainly tasked with Hb degradation. An excess of the 20S proteasome core over the 26S holoenzyme (Anastasiadi et al., 2021b) suggests the dominance of a ubiquitin-independent pathway, which may respond to transient or pathological hypoxia (Xu et al., 2022), although this phenomenon is still being elucidated. The discovery of midnolin, a protein that shuttles non-ubiquitinated nuclear proteins to the proteasome (Gu et al., 2023), may shed some light on this pathway, but it remains to be determined if RBCs contain a midnolin-like protein. Notably, translation- and protein control-related molecules seem to differ in RBCs from different pathophysiological backgrounds, such as with glucose-6-phosphate dehydrogenase deficiency (Tzounakas et al., 2022b), beta-thalassemia trait (Anastasiadi et al., 2021b), and hereditary xerocytosis (Caulier et al., 2022), adding complexity to these “simple” cells.

### Are RBCs nucleic acid-free?

Although devoid of organelles, mature RBCs do contain nucleic acids. For example, circulating RBCs from splenectomized individuals contain nuclear remnants (“Howell-Jolly bodies”) (Angay et al., 2018). Surprisingly, even healthy RBCs contain nuclear and mitochondrial DNA, and higher DNA levels are observed in patients with particular pathology such as cancer; in the latter setting, RBCs seem to take up DNA from cancer cells, although the underlying mechanism needs further investigation (Liang et al., 2023). DNA uptake by RBCs was also reported for cell-free pathogen and mitochondrial DNA bound to the RBC Toll-like receptor 9; thus, under basal, cell-free DNA conditions, RBCs scavenge these deleterious nucleic acids to prevent inflammation (Hotz et al., 2018). Nonetheless, when there are pathological increases in cell-free DNA, as in sepsis or malaria, RBCs undergo changes to sacrifice themselves through erythrophagocytosis, thereby alerting the immune system by presenting CpG-containing DNA (Lam et al., 2021).

Generally, RBCs contain a large variety of RNA molecules, ranging from microRNAs (miRNAs) to mRNAs to large non-coding RNAs (Doss et al., 2015), and this array is altered in disease (Chen et al., 2008, 2017; Sun et al., 2020; Wu et al., 2023). Single-cell transcriptomic analyses revealed that there is even RNA heterogeneity among RBCs from the same subject; in addition, this RNA cargo is also found in RBC-derived extracellular vesicles (EVs) (Jain et al., 2022; Kerkela et al., 2022). Although it is not yet known which of these molecules are merely remnants and which have noncanonical roles, there are some interesting hypotheses. One example is 7SL, the RNA component of the signal recognition particle, which seems to interact with membrane and cytoskeletal proteins in blood samples, including spectrin, band 3, and protein 4.1 (Talhouarne and Gall, 2018). If this interaction is also true for pure RBC populations, it could imply that 7SL RNA is a dynamic component of unknown ribonucleoprotein complexes (Faoro and Ataide, 2021), aiding in the structural integrity of RBCs, or forming potential docking sites. In this view, it is interesting to note that the highly abundant band 3 is characterized by an N-terminus that is extremely negatively charged, thus potentially serving as a docking site for protein-RNA complexes, since RNA-binding proteins are enriched with basic residues to facilitate the interaction with the negatively charged nucleosides. Another noteworthy finding is that EV-secreted miRNAs from RBCs infected with *P. falciparum* may communicate with immune cells (Mantel et al., 2013; Regev-Rudzki et al., 2013). Since erythrophagocytosis can reprogram macrophages (Catala et al., 2020), one may speculate that erythrocytic non-coding RNAs—which have modulatory properties—may also be important in this process. Finally, miRNA composition changes during RBC storage and is associated with the “storage lesion” (Mulatie et al., 2023).

### What if the RBC’s proteome is not that straightforward?

#### Classic RBC proteins with unexpected roles

RBCs possess a complex proteome, the interactome of which is dominated by protein homeostasis, redox biology, and cytoskeletal dynamics (Anastasiadi et al., 2021b; Tzounakas et al., 2021). Even their most well-known and abundant proteins are more multifaceted than was initially anticipated, exhibiting various noncanonical properties. For instance, band 3, the most abundant RBC membrane protein, is an anion transporter and a key component of the junctions between the plasma membrane and cytoskeleton that maintain RBC integrity (Reithmeier et al., 2016). Band 3 is also implicated in the recognition of senescent RBCs by the immune system (Badior and Casey, 2018), but its crucial importance in RBC metabolism was only recently deciphered. Thus, band 3 has a cytosolic chain that serves as a docking site for many molecules, implying its involvement in controlling several processes, ranging from energy and redox metabolism to proteostasis (Issaian et al., 2021; Satchwell and Toye, 2021). The RBC channelome, in general, is of high importance for tightly controlling ion permeability, since a slight flux of cations can alter the normal hallmarks of RBC physiology, such as deformability and shape (von Lindern et al., 2022). Interestingly, apart from its implications in RBC shape and lifespan control (Cahalan et al., 2015), the dynamic regulation of K^+^ transport is essential for maintaining a circadian rhythm in RBCs, in contrast to nucleated cells, which have an intrinsic “clock” mainly based on transcription cycles (Henslee et al., 2017). On another note, glycophorin A (GYPA) provides the RBC surface with a high negative charge, thereby preventing aggregation. GYPA also carries various glycans; given that multiple pathogens use cell surface glycoprotein glycans as receptors for invasion (Imberty and Varrot, 2008), GYPA serves as a potential “decoy receptor.” In this way, pathogens attach to anucleate RBCs instead of their nucleated cell targets (Anderson et al., 2018; Baum et al., 2002; Paulus and van der Hoorn, 2018). Nonetheless, GYPA also acts as a “real” receptor for RBC-targeted pathogens, such as *P. falciparum* (Jaskiewicz et al., 2019).

While known since the 1980s that RBCs contain non-muscle myosin IIA (NMIIA) molecules (Wong et al., 1985), their role was only quite recently deciphered. Although low in abundance, NMIIA contributes to the control of membrane curvature and RBC deformability, through dynamic interactions with actin (Alimohamadi et al., 2020; Smith et al., 2018). Interestingly, during reticulocyte maturation, NMIIA may participate in vesicle clearance (Moura et al., 2018). Because deformability concerns one of the most vital RBC attributes, allowing RBCs to pass through narrow capillaries, multiple methodologies have been used to elucidate RBC flow properties (Artmann et al., 1997; Dao et al., 2003; Guizouarn and Barshtein, 2020). Indeed, elegant microfluidics approaches showed that, after repeated cycles of deformation, an experimental condition that may simulate the accumulated membrane damage during blood circulation, healthy RBCs exhibited mechanical fatigue, which led to a loss of deformability, and increased membrane shear viscosity and energy dissipation, of a magnitude that could cause dissociation of the cell membrane from its cytoskeleton (Qiang et al., 2019). In the same context, when RBCs from healthy controls and, especially, from patients with SCD were subjected to cyclic hypoxic conditions, similar to Hb’s transition from R to T oxygen states, they exhibited reduced deformability (Qiang et al., 2021). However, NMIIA is not the sole contributor to deformability; rather, deformability is a multiparametric phenotype that depends on a wide range of factors, such as RBC hydration, metabolism, structure, and, even, Hb (Huisjes et al., 2018). Thus, it is not surprising that deformability is altered in various pathophysiological contexts and inherited disorders, which underlines its clinical importance. For example, in SCD, the Hb polymerization is the main issue undermining RBC deformability (Huang et al., 2003), whereas, in pyruvate kinase deficiency, decreased deformability is presumably based on insufficient ATP production leading to deregulation of membrane channel function and impaired ion homeostasis (Rab et al., 2021).

*UBB* is seemingly among the top-translated transcripts in mature RBCs and Ub is an important part of the Ub proteasome system (UPS), which is crucial not only for degrading defective proteins but also for regulatory purposes. Ubiquitination of membrane transporters affects RBC capacity to adapt to high altitude hypoxia (Xu et al., 2022) and to pathological hypoxia, as in SCD (Song et al., 2022). For example, degradation of bisphosphoglycerate mutase regulates acclimatization to high altitude hypoxia by constraining synthesis of 2,3-bisphosphoglycerate (Xu et al., 2022)—an allosteric modulator of Hb that promotes oxygen off-loading in response to hypoxia (Webb et al., 2021); however, this same process is deleterious in the context of chronic kidney disease (Xu et al., 2022). Similarly, ubiquitination and degradation of the adenosine equilibrative nucleoside transporter ENT1 (SLC29A1) is deleterious and promotes sickling by boosting bisphosphoglycerate synthesis downstream to adenosine signaling via adenosine A2b receptor (ADORA2B) (Song et al., 2022). Nonetheless, the sheer abundance of the 20S proteasome suggests an additional, non-UPS-related role for Ub in RBCs. Thus, its involvement in establishing protein interactions has been explored primarily in the case of spectrin, which possesses an E2/E3 domain and is simultaneously a target of its activity (Goodman et al., 2015). This domain also targets other important cytoskeletal elements (Chang et al., 2004, 2005), rendering ubiquitination an element in the dynamic regulation of protein associations. For instance, in SCD, the absence of spectrin ubiquitination produces excessively stable cytoskeletal complexes contributing to the characteristic rigidity of irreversibly sickled cells (Chang et al., 2005). Because Ub decorates multiple elements of the RBC membrane-cytoskeleton system, it may function as a scaffolding element. For example, when antioxidant and proteostatic components are translocated to the RBC membrane in response to stress (Anastasiadi et al., 2021b), ubiquitination could promote these interactions.

#### Unanticipated proteins found in RBCs

Surprisingly, multiple unexpected proteins were found in RBCs. As mentioned above, the determination of additional protein synthesis beyond globin chains in mature RBCs has not yet been conclusively ascertained. However, the presence of some reportedly translated transcripts may be plausibly justified, particularly for supporting Hb production. As examples, protein c-Fos and transcriptional factor JunB, members of the activator protein 1 (AP-1) master transcription regulator complex, could be remnants from earlier erythroid differentiation or even serve to enhance transcription of other top-translated genes in RBCs, like globins and *ZFP36*, which all contain AP-1 positive regulatory elements (Loyd et al., 2003). Moreover, zinc finger protein 36 (ZFP36) is itself a translation regulator, through destabilizing mRNAs by binding AU-rich elements (AREs) of their 3’UTRs (Blackshear and Perera, 2014). Because globin genes lack AREs and are stabilized by C-rich elements (Peixeiro et al., 2011; Waggoner and Liebhaber, 2003), one may speculate that ZFP36 facilitates general mRNA degradation in RBCs, thereby favoring the relative enrichment and translation of globin genes.

RBCs also contain members of the gene-regulating nuclear factor kappa B (NF-κB) pathway: NF-κB subunits p65 and p50, and the upstream elements, inhibitor of κB (IκB) and IκB kinase (IKK) (Ghashghaeinia et al., 2011). Treating RBCs with NF-κB pathway inhibitors induces a clearance-related phenotype (Ghashghaeinia et al., 2011), suggesting NF-κB-dependent pro-survival effects. However, these inhibitors also deplete GSH (Ghashghaeinia et al., 2012), and oxidative overload is linked to RBC clearance. It should be noted that, in oxidatively challenged nucleated cells, pathway components (i.e., IκB, IKKB) are oxidized and glutathionylated, inhibiting further pathway activation (Kanayama et al., 2002; Ogino et al., 2005; Reynaert et al., 2006; Seidel et al., 2011), which points to a multi-layer oxidation-driven blockade. Surprisingly, nitric oxide (NO), another negative regulator of RBC clearance, also inhibits IKKB by nitrosylation (Ghashghaeinia et al., 2017; Reynaert et al., 2004). The inhibitory role of glutathionylation and nitrosylation in pathway activation is especially intriguing in RBCs; however, further investigation is needed to determine if the NF-κB pathway is central to multiple RBC lifespan-controlling mechanisms. On another note, in keeping with the presence of RNAs in mature RBCs, proteomics evidence suggests the presence of potentially functional protein machinery for RNA interference silencing complexes, like Dicer (D’Alessandro et al., 2017a) as a remnant of essential miRNA maturation function at the erythroid stage under stress conditions (Byon et al., 2014).

Another surprising RBC cargo is cytokines, a large superfamily of signaling agents. For example, the Duffy Antigen Receptor of Chemokines (DARC; ACKR1) on the RBC surface is involved in the bioavailability of multiple chemokines, including interleukin 8 (Darbonne et al., 1991), small inducible cytokine A5 (RANTES), monocyte chemotactic protein 1 (MCP-1), and growth-regulated alpha protein (GRO-κ) (Horuk et al., 1994; Papadopoulos et al., 2021a). Because the ligand-DARC interaction on RBCs does not initiate intracellular signaling (Neote et al., 1994), RBCs probably function as a cytokine “sink” for sequestering excess circulating cytokines (Fukuma et al., 2003). Although the “sink hypothesis” assumes a passive role for RBCs, an “active reservoir” hypothesis views RBCs as capable of binding and then releasing their pro-inflammatory cargo as needed (Karsten et al., 2018a); the prolonged bioavailability of externally administered DARC-ligands supports this claim (Fukuma et al., 2003). Timely release is another potential function of this reservoir; supporting this concept, macrophage migration inhibitory factor, a highly abundant cytokine, is functionally active and may be released at sites of hemolysis, along with other RBC contents, thereby exerting a pro-inflammatory effect (Al-Abed et al., 2005; Karsten et al., 2018b).

Lastly, α-synuclein, aggregates of which are molecular hallmarks of Parkinson’s Disease, is highly abundant in RBCs (Barbour et al., 2008). It occurs in a monomeric, intrinsically disordered form (Fauvet et al., 2012), which exists in equilibrium with a membrane-associated multimeric form (Bartels et al., 2011). Although it has an affinity for anionic phospholipids, its involvement in membrane regulation was described in several intriguing reports. First, α-synuclein-null mice exhibit mild anemia, with RBCs of reduced volume (Xiao et al., 2014). Moreover, interactions of α-synuclein are not limited to membrane lipids; in *Drosophila melanogaster* neurons, it associates with β-spectrin, disrupting the integrity of the spectrin-ankyrin complex (Maor et al., 2023). It would be useful to explore if these interactions occur in human RBCs and contribute to cytoskeletal disorganization. Finally, Hb also interacts with α-synuclein; this was first identified with neuronal globins and then confirmed in RBCs (Yang et al., 2020).

To date, the mechanism through which protein cargo is transferred to RBCs remains mainly unexplored. Notably, as in the case of nucleic acids, there are indications for protein uptake by RBCs either from other cells or from EVs. As recently shown, mechanical stimulation leads to the formation of distinct temporary cord-like structures between RBCs, which seem to enable protein transfer (Hertz et al., 2023). In the same context, earlier works demonstrated sorting of Band 3 to EVs, as well as its intermembrane transfer from EVs to RBCs (Newton et al., 1983). Analogously, lymphocytes can acquire proteins from other immune cells via trogocytosis (Joly and Hudrisier, 2003). Regarding RBCs, protein transfer mechanisms warrant further investigation; they seem to be strictly regulated by cytoskeletal dynamics because, in most cases, the spectrin-actin network inhibits endocytosis phenomena (Gao et al., 2017).

### What if Hb does not just carry oxygen?

Hb, the most abundant protein in RBCs, contains four subunits, each of which carries a prosthetic heme group. Oxygen binds reversibly to the iron atom of each heme group so that it can be transported to all tissues (Marengo-Rowe, 2006). Nonetheless, oxygen is not the only molecule that binds to Hb. Nitric oxide (NO) can be sequestered by heme, resulting in its low bioavailability and subsequent effects on vasodilation (Helms and Kim-Shapiro, 2013). In contrast, under hypoxic conditions, when endothelial NO production is compromised, Hb can reduce nitrite to NO (Huang et al., 2005) and can also transport NO by conjugation to cysteine thiols in Hb (Jia et al., 1996), highlighting Hb’s role in regulating NO bioavailability. In general, Hb contains docking sites for several molecules, including GSH and NAD(P)H. GSH is critical for RBC antioxidant defenses, and Hb buffers its levels in an oxygen-dependent manner to enhance antioxidant power (Fenk et al., 2022). Similarly, Hb’s NAD(P)H binding and pseudo-enzymatic activities suppress autoxidation and methemoglobin formation (Yamada et al., 2019). These redox-related roles in RBCs perfectly match the observed antioxidant potential of Hb expressed in cancer cells (Li et al., 2013). Hb was additionally proposed to serve as a murzyme, catalyzing ATP synthesis in RBCs (Parashar et al., 2022). Finally, in mice and rabbits, Hb has other unusual functions, acting as a chemosensory signal (Osakada et al., 2022) and antimicrobial molecule (Patgaonkar et al., 2011), respectively. To our knowledge, these latter roles have not yet been reported in humans.

### Is RBC metabolism really that simple?

As highlighted in a recent review (D’Alessandro et al., 2023a), RBC metabolism is far from simple, not only involving glycolysis but also exhibiting an extensive metabolic network, including cytosolic metabolism of tricarboxylic acids (D’Alessandro et al., 2017b), purine (Nemkov et al., 2018b) and arginine (Kalani Roy et al., 2022) metabolism. In addition, RBCs possess receptors and transporters for various important molecules, including insulin, sex and (para)thyroid hormones, catecholamines, neurotransmitters, and multiple drugs (de Almeida and Saldanha, 2010; Gambhir and Agarwal, 1991; Nemkov et al., 2018a; Papadopoulos et al., 2021b). All these render RBCs as a “sink” or transporter for metabolites and signaling molecules, informatively demonstrated in the case of cortisol and aldosterone, both of which seem to be bound and released in a temperature-dependent manner (Papadopoulos et al., 2021b). Activating some of these receptors also induces intracellular signaling cascades. For example, there are indications that shear-induced deformability during blood flow could be regulated by the cyclic AMP/protein kinase A pathway, since inhibitors of this route impair the mechanically induced plasticity of RBCs (Ugurel et al., 2022).

Despite the absence of intracellular compartmentalization in the RBC cytosol, noncanonical metabolism-related microdomains may exist in the RBC membrane (Leo et al., 2020). The most widely discussed such example concerns NO metabolism. Thus, RBCs may possess a “nitrite reductase metabolon” located in lipid rafts and comprised of structural proteins and deoxyHb. DeoxyHb can induce the production of nitrite-derived NO, and the membrane proximity of the implicated reactants and products allows NO to “escape” from Hb’s scavenging properties, and consequently, approach its targets (Gladwin et al., 2005). Additionally, some of the NO produced may react with oxyHb to produce nitrate and metHb, with the latter forming a protective “fence” so that the additional NO produced remains unaffected (Cortese-Krott and Kelm, 2014). RBCs also contain NO synthases (NOS), on the cytoplasmic side of their membrane. Cationic amino acid transporters allow entry of L-arginine into RBCs, which acts as an NOS substrate to produce NO (Cortese-Krott and Kelm, 2014; Gajecki et al., 2022). NOS are also activated during shear stress to regulate vascular properties (Ulker et al., 2009). Taken together, these findings suggest a central role for RBCs in systemic NO metabolism; not just as “pools”, but also as storers, producers, and transporters. Nonetheless, RBCs are not solely mediators in these events, rather NO has been implicated in modulating several RBC properties, including deformability and removal (Brun et al., 2021).

## A dynamic perspective on RBC biomarker and drug targeting potential

**Figure 1.**
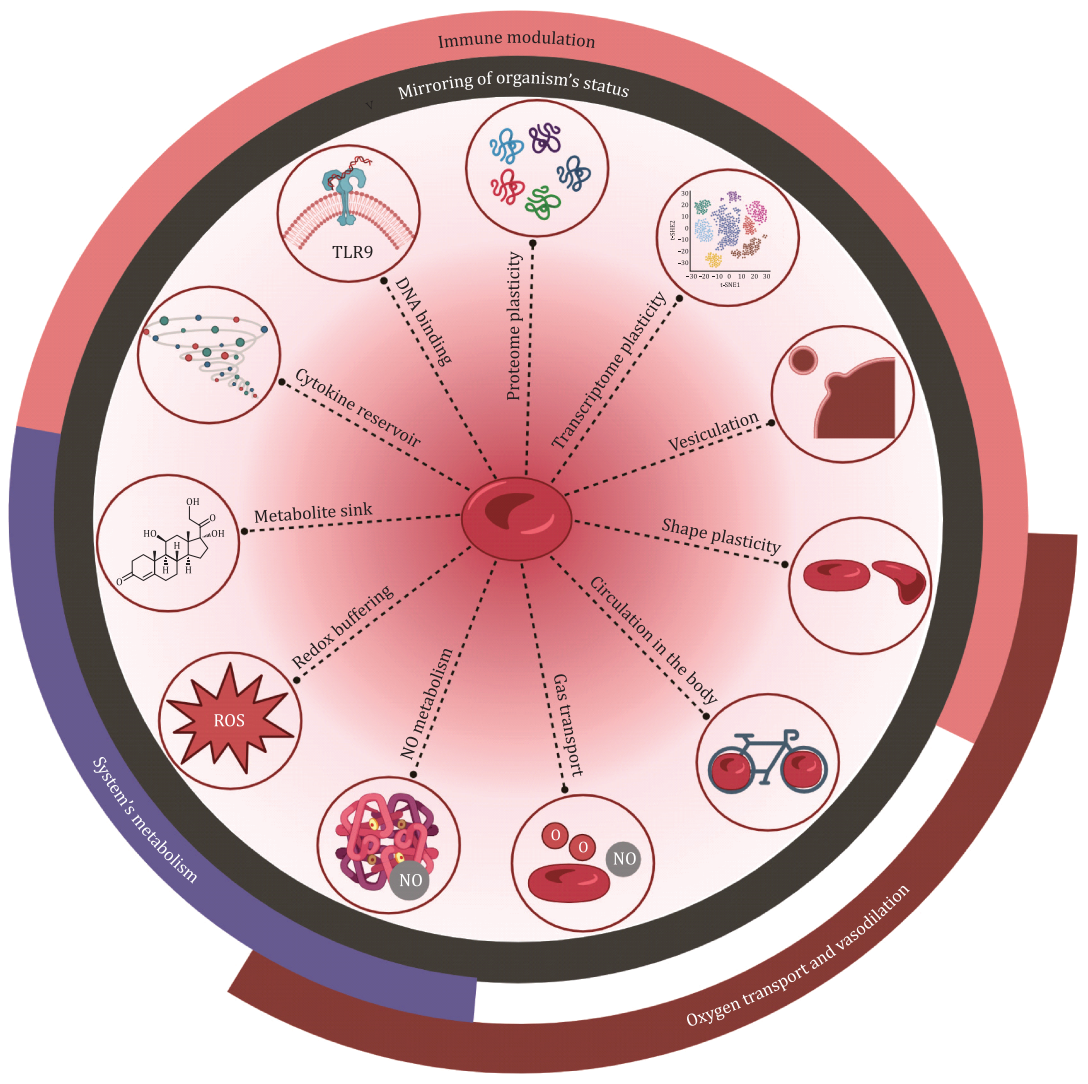
**RBC properties with systemic impact.** The versatile properties of RBCs allow them to reflect an organism’s pathophysiological status; in addition, several of them are crucial for regulating systemic metabolism and vasodilation, and for crosstalk with the immune system. NO: nitric oxide. (Created with Biorender).

The findings discussed above identify that RBCs have a wide array of abilities and functions (Fig. 1). It is noteworthy that numerous intriguing reports describe unexpected RBC cargoes, highlighting the versatility of RBCs. However, we focus on reviewing studies that isolate RBCs by filtration or sorting techniques and/or those that incorporate validation steps to support the purity of the RBCs being examined, as emphasized by the European Red Cell Society (Minetti et al., 2013). Being the most abundant circulating cell type, it is no surprise that RBCs do not just carry oxygen. Thus, RBCs are not simple. Indeed, anucleate RBCs seem to chart a nucleus-independent course that proves advantageous for the entire organism. They can act as redox and ion buffers and monitors of vascular tone. They sacrifice themselves to alert the immune system to “danger.” Thus, they are more than just simple cells, but, rather, appear to function as an “*organ*” (Nemkov et al., 2018a). To date, the focus has been on what RBCs are missing; perhaps it is time to focus on what they have, whether as a remnant, or a vital component, or as something acquired later in their lifespan. Therefore, new information and unexpected findings provide food for thought regarding applications of RBCs in medical settings and lead to a provocative question: What if the “blind watchmaker” (Dawkins, 1986), after years of evolution, has already provided the answer to multiple questions? One answer that is hiding in plain sight, maybe in the form of RBCs.

### RBC disease signatures

Altered RBCs are seen in several pathological conditions and can stratify or characterize patients. For example, the percentage of mitochondria-retaining RBCs in SCD was linked to markers of disease severity, such as sickling and hemolysis (Esperti et al., 2023), whereas RBC mir-144 may be associated with anemia in these same patients. The latter is due to mir-144’s effect on modulating antioxidant powers, through targeting nuclear factor erythroid 2-related factor 2 (NRF2) in RBC precursors, making it a remnant that acts as a useful marker of patients who could benefit from antioxidant treatment (Sangokoya et al., 2010). In the context of transfusion-dependent thalassemia, the deformability of the administered RBCs seems to be a potent indicator of the transfusion outcome, since it has been linked to skin blood flow (Barshtein et al., 2016) and Hb increment (Barshtein et al., 2017). Indeed, transfusion of units containing low levels of rigid RBCs increases the time interval between consecutive transfusions in this patient cohort (Barshtein et al., 2017). Of course, hematological diseases are not the only ones reflected in RBC properties. RBCs can “mirror” the whole organism’s homeostasis due to their constant movement through circulatory networks and interactions with all tissues. The information they collect when traveling through the organism can be “imprinted” on them (Nemkov et al., 2018a), making them useful biomarkers (e.g., glycosylated Hb; HbA1c), especially since blood tests are typically minimally invasive, inexpensive, and easily performed. To support this, RBC distribution width (i.e., RDW) is affected in almost every pathological condition, including hematological diseases (e.g., thalassemia, SCD), and others (e.g., neoplastic, autoimmune, and psychiatric disorders) (Lippi and Plebani, 2014). Therefore, many of the paradoxes discussed above, especially RBCs’ impressive plasticity, may be exploited for diagnostic or prognostic purposes.

**Figure 2.**
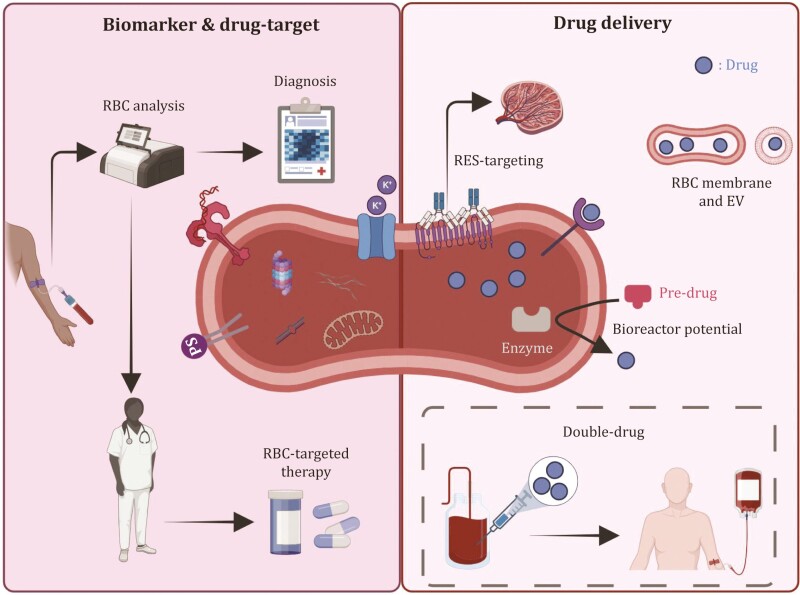
**RBC-based biomarkers, drug targets, and drug delivery potential.** RBCs contain membrane-associated and cytosolic molecules that could be disease biomarkers or drug targets offering timely and accurate prediction, as well as alternative therapeutic schemes. They can also be bioengineered to function as delivery systems to enhance drug administration and extend the circulatory bioavailability of the therapeutic agent. The potential of a scheme that infuses the selected drug attached to the transfused RBCs should not be excluded. RES: reticuloendothelial system, EV: extracellular vesicle. (Created with Biorender).

For example, a liquid biopsy might be useful in early-stage lung cancer patients since their mature RBCs seem to contain copies of DNA with genetic mutations derived from the cancer tissue (Liang et al., 2023). Moreover, the presence of nucleated RBCs might be an independent marker of molecular remission failure in chronic myeloid leukemia (Phan et al., 2019). RBCs are also altered in autoimmunity; in addition to retained mitochondria in RBCs from SLE patients (Caielli et al., 2021), the morphology and biophysical membrane properties of RBCs are altered in rheumatoid arthritis, possibly due to the disease itself or to concomitant factors (Olumuyiwa-Akeredolu et al., 2017). Regarding neurological conditions, and having in mind that (i) α-synuclein interacts with lipid membranes (Sarchione et al., 2021) and (ii) RBCs represent the major source of peripheral α-synuclein, RBC levels of the latter were proposed as a biomarker for Parkinson’s Disease, since α-synuclein blood levels differ between healthy individuals and patients (Abd Elhadi et al., 2019; Tian et al., 2019). Analogously, fibrils on the RBC surface, postulated to be comprised of β-amyloid and tau isoforms, are potential biomarkers for early detection of Alzheimer’s disease (Nirmalraj et al., 2021). One key phenomenon in both Alzheimer’s Disease and RBC responses to hypoxia involves protein isoaspartyl-damage arising from dehydration/deamidation-triggering oxidant challenges (e.g., to structural proteins like 4.1 and band 3 in the aging RBC; to tau protein in Alzheimer’s disease), a process that is, in part, counteracted by protein L-isoaspartyl O-methyltransferase both in RBCs and neural cells (D’Alessandro et al., 2023b). Blood testing to classify patients was recently described using plasma tau protein 217, potentially accurately stratifying and detecting Alzheimer’s disease in a cost-effective way (Brum et al., 2023). Perhaps the most obvious link between neurodegenerative diseases and RBCs is found with the rare neuroacanthocytosis syndromes, such as Chorea-acanthocytosis and McLeod syndrome, in which RBC shape and deformability are altered (Zhang et al., 2013a). Although the mechanism of acanthocyte formation is not completely understood, abnormalities in the levels and conformations of membrane lipids and proteins could be responsible (Kay et al., 1990; Sakai et al., 1991). In children affected by autism spectrum disorder (ASD), a specific spectrum of RBC membranes by using hyperspectral dark field microscopy displayed noteworthy characteristics, since it differed from healthy children and correlated with impaired behavior and cognition scores (Giacometti et al., 2017). In accordance, shape abnormalities were found in RBCs from ASD subjects, without evidence of a specific genetic defect in the progenitor cells (Ciccoli et al., 2013). Moreover, RBCs change during viral infections; for example, their elasticity is reversibly affected when exposed to COVID-19 patient plasma (Recktenwald et al., 2022), whereas senescence markers correlate with disease severity (Bouchla et al., 2021). It does not appear to be a coincidence that activation of antiviral interferon responses in COVID-19 patients is accompanied by metabolic markers such as kynurenine (Recktenwald et al., 2022), fragmentation, and oxidation of structural membrane proteins (Thomas et al., 2020), which in turn have been recently associated to increased osmotic fragility, acute phase response protein deposition on RBC membranes and ultimately increased extravascular hemolysis in healthy, older male blood donors with higher body mass indices (Nemkov et al., 2023). Finally, since RBCs can capture exogenous DNA, they can sequester a detectable amount of cell-free nucleic acids, which would otherwise be undetectable in plasma; this is potentially useful for identifying infectious diseases or cancer at earlier stages. These functions were already leveraged for blood donor surveillance during the Zika virus crisis, showing that negative nucleic acid testing and immunoglobulin M (IgM) to immunoglobulin G (IgG) seroconversion in repeat blood donors infected by this flavivirus still coincided with PCR positivity in mature RBCs for up to ~120 days after infection, the average lifespan of a mature RBC (Catala et al., 2022). Overall, RBC screening could potentially complement currently used serum/plasma biomarkers in various settings, thereby enhancing diagnostic sensitivity and specificity (Fig. 2).

The identification of the potential markers described above was significantly propelled by advances in cutting-edge technologies, such as metabolomics, proteomics, and transcriptomics. Especially in the last decade, the elegant approaches of mass spectrometry and next-generation sequencing were applied to RBCs to construct a more precise “map” of RBC biology and consequently played a pivotal role in unraveling molecular signatures associated with various hematological and non-hematological conditions. Additionally, RBC-specific techniques, particularly those involving deformability, membrane/cytoskeleton mechanical properties, and Hb biochemistry, as well as traditional and state-of-the-art methods for RBC morphology, contributed to the detailed exploration of biomarkers. Nonetheless, the translation of these findings into clinical practice requires solid evidence and careful consideration. Moreover, establishing robust methods and incorporating them into routine clinical settings remains an essential challenge.

### RBCs in therapy

#### RBCs as drug targets

Several disease-related RBC alterations and the unique properties of these cells could serve as potential drug targets (Fig. 2). For example, mitophagy agents in SCD mouse models reduced mitochondrial retention in RBCs and increased RBC lifespan, suggesting a promising therapeutic approach (Jagadeeswaran et al., 2017). Similarly, RBC channel blockers and cAMP-pathway modulators were explored in SCD to counteract cell dehydration and deformability issues, respectively (Ataga et al., 2008; Goskel et al., 2023; Toppet et al., 2000). The new knowledge that RBCs perform low levels of protein synthesis could also be therapeutically useful; thus, drugs enabling stop codon readthrough might be useful for patients with beta-thalassemia who have premature stop codons in their beta-globin gene (Kar et al., 2020; Kumar et al., 2022). Similarly, suggestions of channel transfer in RBCs might provide promising therapeutic interventions targeting channelopathies (Hertz et al., 2023). In addition, in malaria, the protective effect of specific RBC miRNAs (Cyrus, 2021), along with the role of RBC GTPases in the invasion process (Paone et al., 2020), suggest novel drug targets.

Despite our focus in the section above on hematological or RBC-related diseases, RBCs may also be drug targets for multiple other disorders. One example is SLE, in which RBCs are considered a key player due to their mitochondrial cargo (Caielli et al., 2021; Wang, 2021). Additionally, the diminished antioxidant powers of RBCs in multiple sclerosis (MS), the role of RBCs as redox buffers, and the improvement of both RBC redox characteristics and MS symptoms by melatonin supplementation imply that targeting RBCs could be therapeutically useful (Groen et al., 2016). Finally, in cancer, tumors can disrupt normal erythropoiesis, leading to the presence of circulating erythroid progenitor cells (EPC), which could suppress immunity and promote neo-angiogenesis, tumor growth, and metastasis (Zhang et al., 2022). Analogously, the presence of extravascular intratumor mature RBCs, resulting from micro-hemorrhages, can be immunosuppressive by multiple immune-metabolic mechanisms (Papadopoulos, 2022; Papadopoulos et al., 2021a). Taken together, this information suggests using drugs that promote EPC differentiation to mature RBCs along with trying to minimize hemorrhage at tumor sites. One could speculate that such therapeutic schemes can complement currently available oncotherapy, by (i) ameliorating anemia (Madeddu et al., 2021), (ii) improving oxygen delivery to the tumor microenvironment to approximate normoxic conditions, and (iii) reversing suppression of immune cells. Interestingly, polyploid giant cancer cells (PGCCs) can generate their own RBCs that bind oxygen with high affinity due to the expression of fetal and embryonic hemoglobins, providing PGCCs with significant survival advantages (Liu et al., 2022; Zhang et al., 2013b). Further examining tumor erythropoiesis and targeting of PGCCs (or the produced RBCs) are essential to the success of novel RBC-implicating therapeutic anticancer approaches.

#### RBC-based drug delivery

The GYPA decoy theory led to the idea of bioengineering RBCs with decoy viral receptors to prevent invasion of nucleated cells and was examined for several viruses *in vitro* and in animal models (Asher et al., 2005; Hoffmann et al., 2021). However, this is not the only way to bioengineer RBCs, and some have considered them to be ideal drug delivery systems (DDS). Biological DDS are based on natural cells and their derivatives (e.g., RBC ghosts and EVs) and have the significant advantage of biocompatibility. RBCs have been examined as drug carriers since the 1970s and are felt to be useful for this purpose due to their long circulation lifespan, which ensures sustained drug release over time, and their lack of a nucleus and organelles, which provide “space” for drugs (Chen et al., 2023). They can encapsulate therapeutic molecules or carry them on their surface. In the first case, intraerythrocytic agents can either be a non-enzyme drug (e.g. dexamethasone (Chessa et al., 2014)), or an enzyme for replacement therapy purposes. In the latter case, the RBC can function as a bioreactor for removing the accumulated substrate from the bloodstream (Koleva et al., 2020). Two examples include the encapsulation of asparaginase and thymidine phosphorylase, which are proposed for pancreatic cancer and mitochondrial neurogastrointestinal encephalomyopathy patients, respectively (Bax et al., 2019; Hammel et al., 2020; Robert et al., 2022). Regarding surface loading, the binding of glucose derivative-modified insulin on RBCs was reversible in hyperglycemia and prolonged the therapeutic effects of insulin in diabetic mice (Wang et al., 2017). More recently, a scheme of RBC-leveraged chemotherapy was proposed to combat lung metastasis (Zhao et al., 2019, 2021), suggesting a role for RBC-based cancer therapy. Finally, modifying RBCs to expose antigens favoring their opsonization can target them to the reticuloendothelial system, as exploited in studies of cancer and HIV (Pierige et al., 2017), while the target repertoire can be substantially extended by selecting the proper infusion site (Brenner et al., 2018).

Based on RBCs’ intrinsic characteristics and their versatility, RBC-based delivery systems are expected to have broader applications in the future. However, some factors need to be considered before extensive applications in clinical settings. Indeed, during the process of producing RBC-based drug delivery systems, RBCs become less deformable, while the change in the viscoelasticity of their cytoplasm affects cell dynamics (Chen et al., 2023). A decrease in deformability is also observed during surface coupling of therapeutic molecules to the RBC membrane, along with loss of CD47 (integrin-associated protein) and externalization of phosphatidylserine (Villa et al., 2016), all of which can act as signals for rapid clearance from the circulation (Burger et al., 2012; Nguyen et al., 2011; Sosale et al., 2015). For example, attachment of mesoporous silica nanoparticles to the RBC surface impaired their elasticity and deformability (Zhao et al., 2011). Similarly, RBC rigidity may overpower CD47 cell signaling in phagocytosis (Sosale et al., 2015), influence adherence of platelets to the endothelium (Aarts et al., 1984) and, when combined with increased endothelial potential, induce vascular resistance (Kaul et al., 2008). Taken together, these underline the need for strict control of the mechanical properties of RBCs modified for drug transport to extend their circulation time after administration. The pros and cons of using RBCs or their cellular derivatives as drug carriers exceed the scope of the present work and are extensively reviewed elsewhere (Chen et al., 2023; Tzounakas et al., 2017; Villa et al., 2016). Nonetheless, we believe that RBCs will remain a focus of drug delivery innovation.

Of course, RBCs themselves are a “drug” for patients needing transfusion. The variations in donated RBC physiology and biochemistry are relevant to transfusion medicine and are determined by donor genetics, the donor “exposome” (Nemkov et al., 2021), and the ability of RBCs to absorb information from the whole organism. Because stored RBCs from different donors present different redox and metabolism properties, fragility indices, and senescence signals, and also behave differently post-transfusion, these all form the basis for improving personalized transfusion therapy (Anastasiadi et al., 2021a; D’Alessandro and Hod, 2023; D’Alessandro et al., 2021; Thomas et al., 2023; Tzounakas et al., 2022a). Simultaneously, some blood products fail quality control procedures or pass the expiration date before they are administered to patients. Perhaps blood services could apply for a waste management protocol in which blood products that do not meet eligibility criteria for transfusion, including slightly expired or underweight RBC concentrates and fresh frozen plasma from female donors (Tzounakas et al., 2017), could be used for DDS protocols. If this were the case, every single blood drop would be maximized for use for either classic transfusion purposes or alternative therapeutic protocols. Taking this a step further, it would be interesting to produce RBC units loaded with a drug of interest for patients in whom both transfusion and therapeutic interventions were needed, as was proposed for platelet units (Wu et al., 2016). Hence, novel ideas, such as transfusing asparaginase-loaded RBCs in patients with acute lymphoblastic leukemia (Domenech et al., 2011), could possibly be extended to additional disorders.

## We need to keep talking about RBCs

What had started as a basic cell biology and biochemistry quest led to uncovering and rediscovering a multifunctional cell machine with the potential to act as a systemic sensor of disease pathology and as a modulator of physiology. Equipped with a remarkable armamentarium of molecules and cellular properties, RBCs can perform multiple different systemic functions and dedicate their short and strictly controlled lifespan to maintaining organismal homeostasis. Thus, while performing their long-known functions of gas transport and pH buffering in circulation, RBCs can also act as sensors, collecting all sorts of information that are carried by them as extra cargo or as cellular modifications. In this way, we believe that mature RBCs are one of the most promising targets for assessing multiple disease settings, as well as an effective scaffold for designing novel therapies. By applying multi-omics approaches, one can now draw a more complete RBC map and unlock the RBC’s hidden potential to act as a “pathophysiomics” tool itself. Whether the perspectives and opinions provided in this review are biased by the authors’ close encounters with, and appreciation of, this unique cell, or truly represent an opportunity to answer current and future scientific questions, remains to be determined. Until then, we hope you appreciate that “we have a dream” (King, 1963) of an RBC-driven/based biomedicine able to provide new alternatives to health care systems and patients in need.
